# Novel *ANKRD11* gene mutation in an individual with a mild phenotype of KBG syndrome associated to a GEFS+ phenotypic spectrum: a case report

**DOI:** 10.1186/s12881-019-0745-7

**Published:** 2019-01-14

**Authors:** Rita Maria Alves, Paolo Uva, Marielza F. Veiga, Manuela Oppo, Fabiana C. R. Zschaber, Giampiero Porcu, Henrique P. Porto, Ivana Persico, Stefano Onano, Gianmauro Cuccuru, Rossano Atzeni, Lauro C. N. Vieira, Marcos V. A. Pires, Francesco Cucca, Maria Betânia P. Toralles, Andrea Angius, Laura Crisponi

**Affiliations:** 1 Postgraduate Program in Interactive Processes of Organs and Systems - Federal University of Bahia, Salvador, Brazil; 2 Research group Epi-Genétic, Salvador, Bahia Brazil; 3 Centre for Advanced Studies, Research and Development in Sardinia (CRS4), Science and Technology Park Polaris, Pula, Italy; 4 EEG Service and Clinical Outpatient of Epilepsy, University Hospital Complex Professor Edgard Santos (C-HUPES), Federal University of Bahia, Salvador, Bahia Brazil; 5 Department of Biomedical Science, University of Sassari, Sassari, Italy; 6 Institute of Genetic and Biomedical Research, National Research Council (CNR), Cittadella Universitaria di Cagliari, 09042 Monserrato, Cagliari Italy; 7 Clinic Ponto Alto diagnostic by Image, São Marcos, Salvador, Bahia Brazil; 8 Faculty of Medicine of the ABC, São Paulo, Brazil

**Keywords:** Whole exome sequencing, KBG syndrome, *ANKRD11 gene*, Generalized epilepsy with febrile seizures *(*GEFS+), *SCN9A gene*

## Abstract

**Background:**

KBG syndrome is a very rare autosomal dominant disorder, characterized by macrodontia, distinctive craniofacial findings, skeletal findings, post-natal short stature, and developmental delays, sometimes associated with seizures and EEG abnormalities. So far, there have been over 100 cases of KBG syndrome reported.

**Case presentation:**

Here, we describe two sisters of a non-consanguineous family, both presenting generalized epilepsy with febrile seizures (GEFS+), and one with a more complex phenotype associated with mild intellectual disability, skeletal and dental anomalies. Whole exome sequencing (WES) analysis in all the family members revealed a heterozygous *SCN9A* mutation, p.(Lys655Arg), shared among the father and the two probands, and a novel de novo loss of function mutation in the *ANKRD11* gene, p.(Tyr1715*), in the proband with the more complex phenotype. The reassessment of the phenotypic features confirmed that the patient fulfilled the proposed diagnostic criteria for KBG syndrome, although complicated by early-onset isolated febrile seizures. EEG abnormalities with or without seizures have been reported previously in some KBG cases.

The shared variant, occurring in *SCN9A*, has been previously found in several individuals with GEFS+ and Dravet syndrome.

**Conclusions:**

This report describe a novel de novo variant in *ANKRD11* causing a mild phenotype of KGB syndrome and further supports the association of monogenic pattern of *SCN9A* mutations with GEFS+. Our data expand the allelic spectrum of *ANKRD11* mutations, providing the first Brazilian case of KBG syndrome. Furthermore, this study offers an example of how WES has been instrumental allowing us to better dissect the clinical phenotype under study, which is a multilocus variation aggregating in one proband, rather than a phenotypic expansion associated with a single genomic locus, underscoring the role of multiple rare variants at different loci in the etiology of clinical phenotypes making problematic the diagnostic path. The successful identification of the causal variant in a gene may not be sufficient, making it necessary to identify other variants that fully explain the clinical picture. The prevalence of blended phenotypes from multiple monogenic disorders is currently unknown and will require a systematic re-analysis of large WES datasets for proper diagnosis in daily practice.

## Background

Whole Exome Sequencing (WES) technology introduces a remarkable revolution in the identification of disease-causing genes and a powerful tool for genetic diagnosis, mainly relevant for rare diseases, which is remarkably difficult for clinicians to be diagnosed. WES can substantially reduce the number of cases remaining undiagnosed for many years and has become the current standard for the diagnosis of highly heterogeneous rare disorders with suspected Mendelian inheritance. This approach is leading to better dissecting the clinical phenotype of patients, in particular, those related to phenotypic progression in association with a single locus and those derived from mixed phenotypes developing from multilocus genomic variants. Recent studies reported the presence of multiple genomic diagnoses in a single individual in 3.2–7.2% of cases [1–3].

Molecular variants in the SCN9A gene (MIM#603415) are responsible for a range of seizure disorders, which are characterized by early-onset isolated febrile seizures to generalized epilepsy with febrile seizures plus, type 7 (GEFS+), which identifies the most severe phenotype, as well as primary erythermalgia, callousness to pain linked to channelopathy and extreme paroxysmal pain disorder. Patients with isolated febrile seizures frequently showed an early onset between ages 5 months to 4 years and exhibit natural remission by age 6 years, while patients with GEFS+ persist in having various types of febrile and afebrile crises afterwards in life [4]. The *SCN9A* gene encodes for NaV1.7, a voltage-gated sodium channel mainly expressed in the hippocampus during the embryonic phase, suggesting a key function in the central nervous system [5] and in nociception signaling. *SCN9A* has been proposed suggested as a genetic modifier in *SCN1A* mutation linked with GEFS+ and as a potential susceptibility gene for Dravet syndrome [6, 7].

In 1975, KBG syndrome was identified and characterized by specific clinical findings: macrodontia of the upper central incisors, distinctive craniofacial signs, short stature, skeletal abnormalities and neurological involvement that encompasses developmental delay, convulsions and intellectual disability [8–11].

The initial description of the KBG syndrome, named KBG based on the initials of first affected families’ surnames, referred to 7 patients from 3 unrelated families with a putative autosomal dominant inheritance [8, 12, 13]. To properly diagnose KBG syndrome, 4 or more of these 8 major criteria should be satisfied: (1) macrodontia of the upper central incisors, observed as a distinctive trait of KBG syndrome and reported in more than 95% of cases; (2) distinctive facial features (presence of at least three findings of six categories of craniofacial shape, hair/eyebrow, eyes, ears, nose and mouth); 3) hand abnormalities, (fifth finger clinodactyly, clinical brachydactyly, or short tubular bones on radiographic exam); (4) neurological implication, with magnetic resonance, global developmental delay, and/or a seizure disorder; (5) bone age > 2 SD below the average; (6) costovertebral abnormalities, (abnormal curvature of the spine, cervical ribs, or vertebral/endplate defects); (7) postnatal short stature (as a height less than 3rd centile); and (8) occurrence of a first-degree relative affected by KBG syndrome.

In 2011, Sirmaci et al., [14] identified pathogenic heterozygous variants in the *ANKRD11* gene (MIM# 611192). Single nucleotide mutations and small indels represent about 83% of the pathogenic variants identified within ANKRD11 and larger copy number variants (mostly deletions) represent about 17% [15–17].

ANKRD11 represents one of the family members of ankyrin repeat-containing cofactors that relates with p160 nuclear receptor coactivators (NCOA1) by recruiting histone deacetylases to inhibit ligand-dependent transcriptional activation [14, 18, 19]. ANKRD11 was also found to localize inside the neurons nuclei and to accumulate in distinct inclusions after their depolarization. This finding suggests that ANKRD11 represents one of the major players in neural plasticity [14].

Here, we report two sisters from a non-consanguineous family, presenting generalized epilepsy with febrile seizures plus (GEFS+; MIM#613863) associated with an heterozygous mutation in the *SCN9A* gene, p.(Lys655Arg), inherited from the father that was asymptomatic for the crisis, and a novel de novo loss of function mutation in the exon 10 of the *ANKRD11* gene, p.(Tyr1715*) in one sister with a clinical phenotype compatible with KBG syndrome (MIM#148050).

## Case presentation

### Clinical data

The reported family comes from Brazil and the two probands share a GEFS+ phenotypic spectrum. The original purpose of our study was to find the molecular causes of such phenotype. All members of the family were clinically assessed and diagnosed by the respective clinical neurologist and geneticist.

### Case II-1

**Fig. 1:**
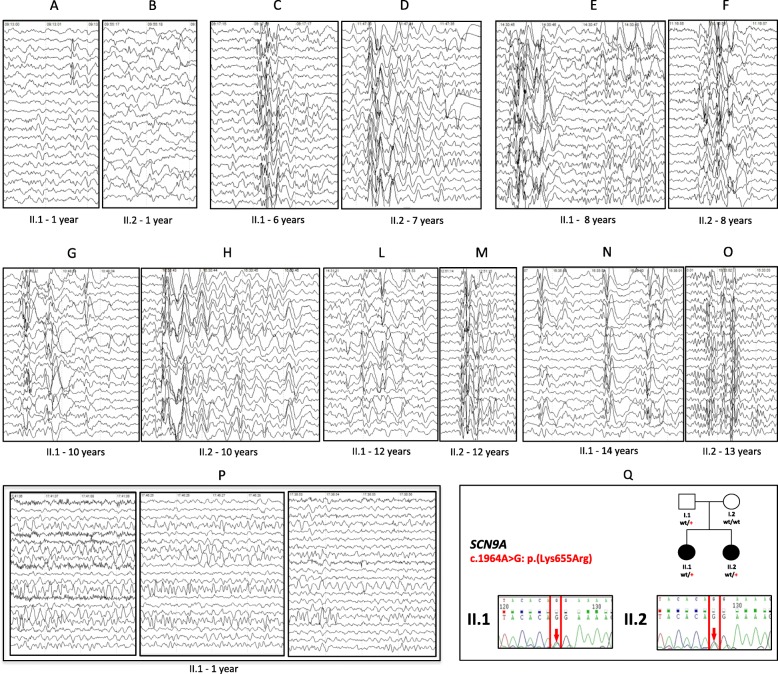
Electroencephalogram (EEG) and mutation status of the *SCN9A* gene in cases II.1 and II.2 associated to GEFS+ phenotypic spectrum. **a**-**p**: EEG evolution of the two sisters starting from 1 to 13–14 years of age. P: Theta rhythm (4-5 Hz) in the temporo-occipital regions (T5-O1; T6-O2) in case II.1, at 1 year of age. **q**: Pedigree of the family under study. Mutation status of the *SCN9A* gene is indicated beneath symbols for each subject. Sanger sequencing of cases II.1 and II.2: arrow indicates the presence of the c.1964A > G: p.(Lys655Arg) mutation

Female 14.5 years old. Delivered full term, by emergency C-section, due to lack of fetal movement, weighing 2.971 Kg, 48 cm long and 33 cm head circumference. Neonatal period had no complications. From the neonatal period onward, she showed difficulty to breastfeed, with low weight gain. At 9 months old, she had myoclonus-atonic type seizures with sudden falling of the head and trunk. Initially precipitated by fever, these seizures became afebrile and daily, several times a day, and were controlled after substituting phenobarbital for sodium valproate (VPA), in low doses. The EEG tests initially showed focal spikes (centro-temporal regions) and only at 4 age, one EEG test showed a theta rhythm (4-5 Hz) in the temporo-occipital regions (T5-O1; T6-O2). At the age of 4 years and 8 months, after remission of seizures for 3 years, and normal EEG tests, VPA was suspended. Starting from 6 years of age, the EEG tests showed persistence of several bursts of irregular generalized polyspike-wave (PSW) and spike-wave discharge (SW), lasting 1–3 s. (Fig. 1a-p). Despite persisting abnormal EEGs, patient has not presented relapse of seizures and is not on medication.

**Fig. 2:**
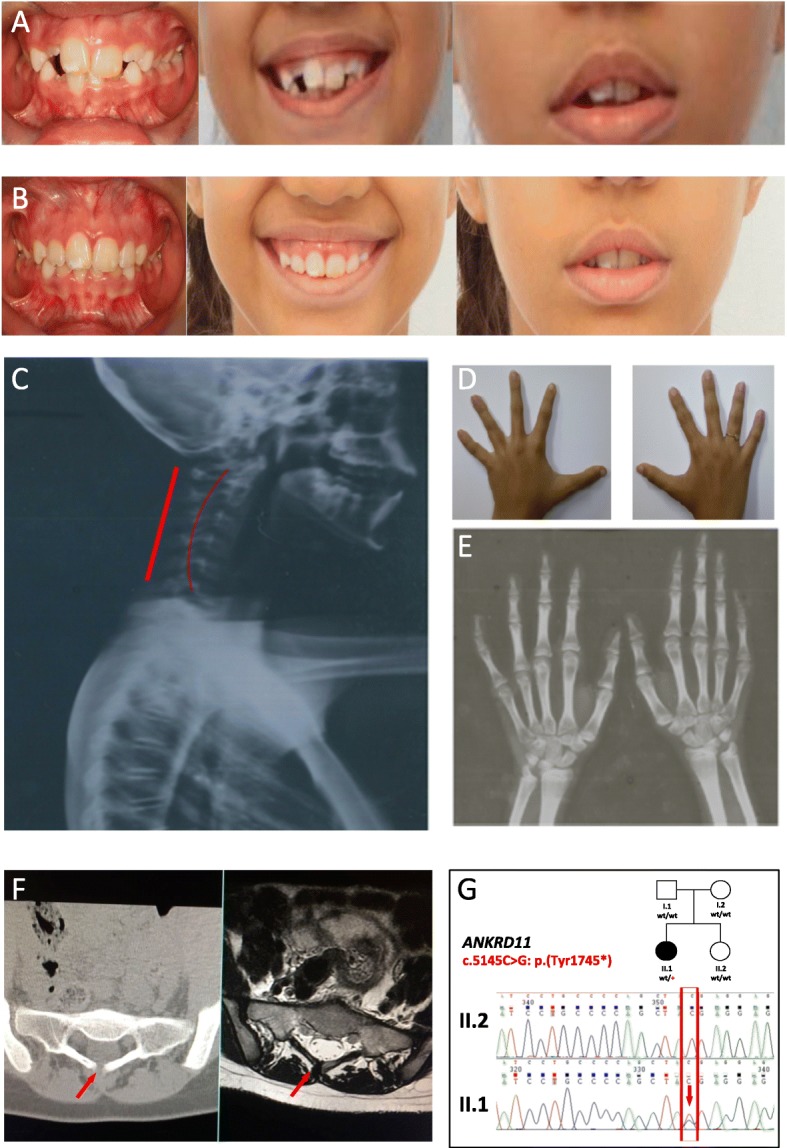
Clinical features of case II.1 carrying the *ANKRD11* mutation associated to the KBG syndrome. **a** and **b**: intra and extra oral views at 8 and 10 years, respectively. Due to the dental apparatus (an expander to enlarge the palate positioned at the age of about 8 and a half years), it was not possible to confirm the patient’s sub-mucosal palate with magnetic resonance imaging. The clinical suspicion is based on the dentist’s assessment of the palate. **c**: X-rays view of the cervical spine presenting invasion of physiological cervical lordosis. **d** and **e**: Picture and X-rays of patient hands demonstrating shortening of the distal phalanx of the 5th finger, clinodactyly of the 2th and 5th with a slight shortened tubular bones III and IV. **f**: Computed tomography and magnetic resonance imaging of the sacroiliac joints that allowed the display of concealed spina bifida at L5/S1. **g**: Pedigree of the family under study. Mutation status of the *ANKRD11* gene is indicated beneath symbols for each subject. Sanger sequencing of cases II.1 and II.2: arrow indicates the presence of the c.5145C > G: p.(Tyr1745*) mutation

Patient presented with recurrent otitis episodes and developed conductive hearing loss in left ear. A computed tomography scan of the mastoid showed signs of otomastoiditis in the left ear with obliteration of Prussak’s space and cholesteatomatous process. Orthodontic evaluation conducted at 8 years of age showed dolichofacial pattern, maxillary protrusion, absence of lip seal, delayed eruption of permanent teeth, besides size increase of upper central incisors, with extra mamelar structures and whitish material of incisors and other teeth, compatible with hypoplasia (Fig. 2a, b). Cone-beam computed tomography of right oral lower-posterior region at 14.5 years of age, revealed dental units partially erupting and the presence of mixed-aspect images located between the dental roots, suggesting bone dysplasia (Fig. 2). The skeletal X-ray assessment showed inversion of physiological cervical lordosis (Fig. 2c); deviation of left dorsal axis, accentuated thoracic and lumbar lordosis and concealed spina bifida at L5/S1 (Fig. 2f). The proband has also shortening of the distal phalanx of the 5th finger, clinodactyly of the 2th and 5th (Fig. 2d, e); myopia; bifid uvula with submucous cleft palate; weight and height growth curve below percentile < 5. Neuropsychological analysis at age 8 showed IQ of 73.

### Case II-2

Female 13 years old. Showed normal neurological development. At 12 months, she had the first febrile seizure. She had recurring febrile seizures kept under control with low doses of VPA. After 3.8 years with no relapse of seizures and normal EEGs, VPA was suspended. At age 8, the same electroencephalographic pattern observed in her sister appeared on the EEG (Fig. 1 a-p), with persistence of bursts of irregular generalized polyspike-wave (PSW) and spike-wave discharge (SW), less frequent and with shorter extent, with no relapse of seizures and no medication. She presents normal neuro-psychomotor and weight-height development, and an absence of dysmorphic and radiologic alterations.

### Genetic analysis

Peripheral blood lymphocytes were collected from all affected individuals and their parents, and genomic DNA was extracted using a salting out procedure [20].

An array based Comparative Genomic Hybridization (CGH) analysis was done using commercially available Human Genome CGH Microarray (Agilent Technologies, Waldbronn, Germany) with an estimated average resolution of 13Kb (SurePrint G3 Human CGH Microarray).

NGS panel analysis was performed by Ampliseq/Ion Torrent technology with at least 120X using a targeted re-sequencing of 21 genes implicated in juvenile forms of epilepsy (ARHGEF9, KCNQ2, PRRT2, PNKP, ST3GAL3, SCN1A, GRIN2A, SCN8A, SLC2A1, SPTAN1, SCN2A, ALDHTA1, PCDH19, ARX, TBC1D24, KCNT1, PLCB1, STXBP1, PNPO, CDKL5, SLC25A22). No pathogenic variant has been identified with this method in our siblings.

One hundred nanograms of genomic DNA was used for DNA library preparation and exome enrichment using the Nextera Rapid Capture Expanded Exome Kit (Illumina) according to manufacturer instructions. DNA1000 chips (Agilent) and Qubit dsDNA BR Assay Kits (Invitrogen) were used to assess the quality of libraries. An indexed paired-end sequencing run (101 + 7 + 101 bp) was performed on a HiSeq 2000 using SBS Kit v3 chemistry (Illumina).

Using an analysis pipeline implemented in Orione [21], we performed read alignment to the human reference genome (hg19) using the Burrows-Wheeler Aligner 7 (BWA-MEM; version 0.7.5a) and GATK framework (version 2.8.1). Using GATK Unified Genotyper and GATK Variant Annotator modules, we annotated the variants as known or novel based on dbSNP146 and SnpSift/ SnpEff and KGGSeq. We used different models (SIFT, Polyphen2, LRT, MutationTaster, MutationAssessor and FATHMM) to assess the functional predictions for the aminoacid changes. We filtered the identified variants according to recessive/dominant/de novo pattern of inheritance, gene features and MAF < 1% using as references dbSNP138, dbSNP141, 1000 Genomes, ESP6500, ExAC, gnomAD and EVADE, our private database of about 600 exomes). Subsequently, variants were evaluated for their phenotypic and biological impact.

The average target coverage was 93.5, 83.6, 88.1 and 78.9 for father, mother, case II-1 and case II-2, respectively. The target region was covered at least 10X in 93% for the father, 92% for the mother, 93% the older sister and 92% the younger. After filtering of the variants and the quality assessment in IGV browser, we identified a *SCN9A*: NM_002977:c.1964A > G: p.(Lys655Arg) (rs121908919); chr2:167138296 T/C in hg19 variant in both cases, inherited from an unaffected father. Additionally, we found a novel de novo truncating mutation in exon 10 of the *ANKRD11* gene: NM_001256182:c.5145C > G: p.(Tyr1715*); chr16:89347805 G/C in hg19 in case II-1 (Figs. 1 and 2).

## Discussion and conclusions

We report here a dual diagnosis in case II-1 presenting with *ANKRD11* and *SCN9A* pathogenic variants found by WES. The variant in *ANKRD11* has never been reported so far in literature. Considering that it is de novo, we supposed a possible mosaicism in the parents but we were not able to find any by WES analysis. The reassessment of phenotypic features in case II-1 confirmed that she fulfilled the proposed diagnostic criteria for KBG syndrome, complicated by early-onset isolated febrile seizures, although EEG abnormalities with or without seizures have been reported in some KBG cases [17]. In particular, she presented with macrodontia, hand anomalies, neurological involvement, costovertebral anomalies and post-natal short stature. This is the first Brazilian case reported so far.

The KBG syndrome is very rare, with about 100 individuals reported so far in literature [15, 16]. Probably it is underdiagnosed due to the clinical features that can be mild and common to other diseases. ANKRD11 mutations have been found in patients identified in a large number of subjects with characteristics consistent with Cornelia de Lange syndrome (see, for example, CDLS1 MIM#122470), thus showing phenotypic overlap between the two disorders. As previously reported [22, 23], some KBG patients could be recognized by gestalt, others may look like Cornelia de Lange syndrome (CDLS). CDLS and KBG represent two rare and distinct syndromes, but they have clinical aspects that overlap such as cognitive deficit, growth retardation and certain craniofacial abnormalities (brachycephaly, wide eyebrows and nostrils anti-vertite). Other common features are limb abnormalities such as small hands and feet, clinodactyly of the fifth finger and syndactyly of the second and third toes. Five different genes associated with the cohesin complex and its regulation (*NIPBL, SMC1A, SMC3, HDAC8* and *RAD21*), showed heterozygous mutations identified in patients with CDLS. The cohesin complex regulate gene expression mediating transcriptional activation and repression [23]. The main function of ANKRD11 is to suppress transcriptional activation of the target genes of nuclear receptors by enrolling deacetylase at different promoters [18]. It is reasonable to assume that dysregulation of functionally correlated genes by cohesin complex deficiency or ANKRD11 may result in overlapping phenotypic characteristics [23].

The shared variant p.(Lys655Arg), occurring in *SCN9A,* has been previously found in several individuals: one with GEFS+ and two with Dravet syndrome. Additionally, one of these individual also possesses a de novo SCN1A mutation [6] and another affected by atypical benign partial epilepsy (ABPE) of childhood, harbored variants in in the CPA6 and SCNM1 genes associated with epilepsy [24]. Although already shown to play important pathogenic roles in epilepsy and predicted to significantly alter protein function, the p.(Lys655Arg) variant was detected in asymptomatic parents and has been found with extremely low frequency in control cohorts (NHLBI GO Exome Sequencing Project and ExAC Browser).The SCN9A p.Lys655Arg variant found in our sisters seems to be associated with an early-onset isolated febrile seizures. Other subjects showing febrile seizures or GEFS+, that possess *SCN9A* mutations were sporadic and cannot offer powerful evidence for a specific role of *SCN9A* in seizure disorders that is presently under debate. Therefore, this report would further support that *SCN9A* mutations are linked to a monogenic pattern.

The 39-year-old father, carrier of the same *SCN9A* variant, has not reported any history of seizures. Considering that most febrile seizures would naturally remit with age, we reinvestigated the family but we did not find evidence of history of seizure disorders in the father. However, he has had attention-deficit hyperactivity disorder since childhood and his family history shows neuropsychiatric disorders (dementia, schizophrenia, personality disorder, panic disorder, depression, delayed development, intellectual disability and autism spectrum disorder).

The phenotype of patients with GEFS+ combine febrile seizures, absence seizures, partial seizures, myoclonic seizures, or atonic seizures, with a variable degree of severity [25]. In a recent paper [7], none of the GEFS+ families analyzed could be completely clarified by high penetrance of SCN9A mutations. Moreover, it is not surprising to identify a non-penetrant individual because they are commonly identified in autosomal dominant diseases and well documented in pedigrees of febrile crises (60–80% of penetrance [6]).

This study provides an example of how WES has been instrumental allowing us to dissect the clinical phenotype, which is a multilocus variation aggregating in one proband. The successful identification of causal variant in a gene may not be sufficient, making it necessary to identify other variants that can fully explain the clinical picture. In several series of studies, the presence of multiple molecular diagnoses in a single individual has been described in 3.2–7.2% of cases that underwent molecular analysis, but large cohorts of patients and its associated clinical studies [3] are lacking to clearly define this phenomenon. This report emphasizes the critical role of the clinician in diagnostic genomic analyses and highlights the advantages of WES technology in the genetic dissection of a heterogeneous phenotype.

In our study, we prove that apparent phenotypic expansion may represent blended phenotypes resulting from pathogenic variation at more than one locus, so allowing a dissection of genotype–phenotype relationships. Due to the lack of the prevalence of blended phenotypes from multiple monogenic disorders, a systematic re-analysis of WES data sets is needed to a proper diagnosis in daily practice.

## Abbreviations

ANKRD1: Ankyrin Repeat Domain 11
Array based CGH: Microarray based comparative genomic hybridization
CDLS: Cornelia de Lange syndrome
CPA6: Carboxypeptidase A6
EEG: electroencephalogram
GEFS: generalized epilepsy with febrile seizures
HDAC8: Histone Deacetylase 8
IQ: intelligence quotient
KBG: syndrome
L5/S1: Lumbar5/Sacral1 Junction
NaV1.7: Nav1.7, sodium channel encoded by SCN9A
NCOA1: nuclear receptor coactivator 1
NGS: Next Generation sequencing
NIPBL: Nipped-B-like protein gene
PSW: polyspike-wave
RAD21: RAD21 Cohesin Complex Component
SCN9A: Sodium Voltage-Gated Channel Alpha Subunit 9
SCNM1: Sodium channel modifier 1
SMC1A: structural maintenance of chromosomes 1A
SMC3: Structural maintenance of chromosomes protein 3
SW: spike-wave discharge
VPA: sodium valproate
WES: Whole exome sequencing
